# Draft genome sequences of five *Lactobacillus gasseri* strains isolated from voided urine samples

**DOI:** 10.1128/mra.01119-23

**Published:** 2023-12-22

**Authors:** Natalie Stegman, Maria Steiling, Cerena Sedano, Briana Jackson, Catherine Putonti

**Affiliations:** 1 Bioinformatics Program, Loyola University Chicago, Chicago, Illinois, USA; 2 Department of Biology, Loyola University Chicago, Chicago, Illinois, USA; Department of Biological Sciences, Wellesley College, Wellesley, Massachusetts, USA

**Keywords:** *Lactobacillus gasseri*, urinary tract, urobiome

## Abstract

*Lactobacillus gasseri is* a member of the gut, oral, and female urogenital microbiota. Here, we present the draft genome assemblies of *L. gasseri* UMB1549, UMB1579, UMB1644, UMB3348, and UMB5890, which were isolated from voided urine samples from females with Type 2 diabetes.

## ANNOUNCEMENT

*Lactobacillus gasseri* is found in multiple anatomical sites of the human body (1–4). In rodent models, it has been shown to control Type 2 diabetes development and improve diabetic symptoms (5, 6). *L. gasseri* strains have been studied for their antimicrobial effects, including bactericidal activity against urogenital pathogens (7). Thus, we have been isolating *L. gasseri* strains from urine to further our understanding of their antimicrobial properties in the urinary tract (8).

Here, we present the draft genomes of five *L. gasseri* strains: UMB1549, UMB1579, UMB1644, UMB3348, and UMB5890. All strains were isolated from voided urine samples from females with Type 2 diabetes (Loyola University Chicago IRB# 204197) (9) via the enhanced quantitative urine culture method as part of a prior study (10). Species designations were confirmed via matrix-assisted laser desorption/ionization time-of-flight (MALDI-TOF), as previously described (11), and stored at −80°C in the Loyola Urinary Education and Research Collaborative (LUEREC) collection. We obtained the freezer stocks from the collection and streaked them on deMan-Rogosa-Sharpe (MRS) agar plates and incubated with 5% CO_2_ at 35°C for 48 hours. Then, a single colony was grown in MRS medium supplemented with 1  mL/L of Tween 80 (Sigma-Aldrich) and incubated with 5% CO_2_ at 35°C for 48 hours. DNA was extracted using the DNeasy Blood and Tissue Kit (Qiagen) following the manufacturer’s protocol for Gram-positive bacteria and quantified using a Qubit fluorometer.

DNA was sent to SeqCenter (Pittsburgh, PA, USA) for library preparation and sequencing. Libraries were created using the Illumina DNA Prep kit and custom IDT 10 bp unique dual indices with a target insert size of 320 bp and sequenced using the Illumina NovaSeq 6000 platform (2 × 151 bp paired-end reads). Raw reads were trimmed using BBDuk, which is part of the BBMap suite (v39.01; sourceforge.net/projects/bbmap/) with the following parameters: qtrim = rl, ftl = 15, ftr = 135, maq = 20, maxns = 0, and trimq = 20. Trimmed reads were assembled using SPAdes v15.5 with the --only-assembler option (12). Genome coverage was calculated using BBWrap (also part of the BBMap suite). Genome assemblies were annotated upon submission to the NCBI by the Prokaryotic Genome Annotation Pipeline v6.6 (13).

Table 1 lists genome sequencing and assembly information for all five strains. Average nucleotide identity (ANI) was computed for each assembly using JSpeciesWS and the genome *L. gasseri* PSS7772D (14). All five strains had an ANI value greater than or equal to 98.87, confirming their species assignment.

**TABLE 1:** Genome sequencing and assembly statistics of urinary *L. gasseri* strains

UMB	No. of raw reads	No. of contigs	Length of the genome (bp)	Contig *N*_50_ (bp)	GC content (%)	Coverage	SRA accession	GenBank assembly accession
UMB1549	2,543,588	59	1,981,741	59,794	34.80	186.507×	SRR26087531	GCA_032377135.1
UMB1579	4,354,262	59	1,979,332	59,403	34.90	301.829×	SRR26087539	GCA_032432775.1
UMB1644	3,537,846	62	1,868,268	65,753	34.90	269.925×	SRR26087538	GCA_032432735.1
UMB3348	2,930,412	15	1,787,658	357,091	34.90	238.284×	SRR26087536	GCA_032377295.1
UMB5890	2,894,018	61	1,977,892	54,253	34.90	211.343×	SRR26087537	GCA_032432755.1

To investigate their putative antimicrobial activity, the genome assemblies were screened for bacteriocins and ribosomally synthesized and post-translationally modified peptides using Bagel4 (15). All five strains encode for the heat-sensitive bacteriocin helveticin (16). Furthermore, UMB1549, UMB1579, UMB1644, and UMB5890 encoded for acidocin B (17). *L. gasseri* UMB5890 also encoded for pediocin. The genome annotations warrant further testing of these strains to determine if they produce bacteriocins and/or have antimicrobial effects.

## Data Availability

Raw reads and draft assemblies have been deposited in GenBank. Table 1 lists the SRA accession numbers and genome assembly accession numbers.
